# Cost-Effective Fabrication of Silica–Silver Microspheres with Enhanced Conductivity for Electromagnetic Interference Shielding

**DOI:** 10.3390/nano15181433

**Published:** 2025-09-18

**Authors:** Mingzheng Hao, Zhonghua Huang, Wencai Wang, Zhaoxia Lv, Tao Zhang, Wenjin Liang, Yurong Liang

**Affiliations:** 1 The Department of Materials Engineering, Taiyuan Institute of Technology, Taiyuan 030008, China; haomz@tit.edu.cn (M.H.); lvzx@tit.edu.cn (Z.L.); zt15757609630@163.com (T.Z.); 718lwj@gmail.com (W.L.); 2 Shanxi Center of Technology Innovation for Polyamide Materials, Taiyuan 030008, China; 3 Unit 93129 of the Chinese People’s Liberation Army, Beijing 100843, China; hzh710903@sina.com; 4 Key Laboratory of Beijing City on Preparation and Processing of Novel Polymer Materials, Beijing University of Chemical Technology, Beijing 100029, China

**Keywords:** silica microspheres, silver coating, surface modification, electrical conductivity, electromagnetic interference shielding

## Abstract

A green and cost-effective method was employed to efficiently synthesize conductive silica–silver (SiO_2_/PCPA/Ag) core–shell structured microspheres. The SiO_2_ microspheres were initially functionalized with poly(catechol-polyamine), followed by the in situ reduction of Ag ions to Ag nanoparticles on the surface of the SiO_2_ microspheres using an electroless plating process. Analysis using scanning electron microscopy confirmed the successful formation of a dense and uniform silver layer on the surface of the SiO_2_ microspheres. The valence state of the silver present on the surface of the SiO_2_ microspheres was determined to be zero through analyses conducted using an X-ray photoelectron spectrometer and X-ray diffractometer. Consequently, the SiO_2_/PCPA/Ag microspheres, upon initial preparation, demonstrated a notable conductivity of 1005 S/cm, which was further enhanced to 1612 S/cm following additional heat treatment aimed at rectifying defects within the silver layer. The resulting rubber composites displayed a low electrical resistivity of 5.4 × 10^−3^ Ω·cm and exhibited a significant electromagnetic interference (EMI) shielding effectiveness exceeding 100 dB against both X-band and Ku-band frequencies, suggesting promising potential for utilization as a material for conducting and EMI shielding purposes.

## 1. Introduction

The increased utilization of electrical and digital devices in industrial, commercial, and military sectors has resulted in electromagnetic interference (EMI) emerging as a significant concern in contemporary times. EMI has the potential to cause malfunctions in delicate electronic components and pose harmful effects on living organisms [1,2,3]. In order to address this issue, extensive research has been conducted on electromagnetic shielding materials, including metals [4,5], carbon-based materials [6,7], and electrically conductive polymers [8,9,10]. Nevertheless, these materials have certain drawbacks that hinder their widespread adoption. Metals exhibit high density and susceptibility to corrosion [11,12], while carbon materials are prone to oxidation at low temperatures [13]. Electrically conductive polymers are currently costly, difficult to manipulate, and necessitate significant improvements in mechanical properties [14,15]. Consequently, there is an urgent need for electromagnetic shielding materials that offer superior shielding effectiveness, lightweight construction, satisfactory mechanical characteristics, and cost-effectiveness.

Metal-encapsulated micro/nanoparticles continue to garner significant interest for their potential applications in various fields, including catalysis, magnetics, electronics, and photonics [16,17,18]. Silver is commonly utilized in electromagnetic shielding materials due to its excellent electrical properties. Silica microspheres, characterized by their sphericity, low density, non-toxicity, cost-effectiveness, and high strength, have been widely employed as a raw material for the production of lightweight core–shell composites [19]. Numerous methods have been documented for the preparation of silver-coated silica microspheres, such as sputtering methods, in situ chemical reduction, layer-by-layer self-assembly, and electroless plating [20,21,22]. Electroless plating is a preferred technique due to its ease of equipment, cost-effectiveness, and compatibility with various substrates. However, the lack of active functional groups on the surface of silica often results in unsatisfactory adhesion between the silver layer and silica microspheres. Therefore, surface pretreatment or modification is essential to enhance the adhesion between silver and silica. The conventional sensitization–activation method, while commonly used, has drawbacks such as laborious processes, environmental concerns, and inconsistent coating quality, limiting its practical applicability. Various surface modification techniques have been employed for the preparation of silver-coated silica, including sol–gel [23], silane coupling [24], and layer-by-layer assembly [25]. However, these methods are associated with drawbacks such as volatile organic compound emissions and complexity. Therefore, a straightforward, adaptable, and environmentally friendly pretreatment or modification of the substrate is essential to address these issues.

It has been reported that poly(dopamine) can be deposited on a wide range of natural and inorganic materials through self-polymerization of dopamine in an alkaline aqueous solution at room temperature [26]. This method offers advantages such as simple components and equipment, moderate reaction conditions, environmental friendliness, and versatility in its application to various materials. Additionally, the poly(dopamine) layer can serve as a versatile platform for subsequent reactions [27]. The metal-binding capability of catechol and the reducing capacity of the amine group in the poly(dopamine) structure have been utilized to achieve uniform, compact, and continuous metal layers on a range of substrates in our previous work, including microspheres, fibers, and carbon nanotubes.

However, the high cost of dopamine and low formation rate of the poly(dopamine) layer have hindered the widespread practical application of dopamine. In response to this limitation, Wang et al. [28] proposed a more cost-effective alternative using a binary system of catechol and polyamine to replace dopamine, resulting in the production of a hydrophilic PP separator. This approach, which is significantly more affordable (at only 8% of the cost of dopamine), has proven to be efficient and versatile. Given that the catechol/polyamines share the same functional group as dopamine, we speculate that this poly(catechol-polyamine) modification method can also be used for surface metallization of materials.

In this study, the surface of silica microspheres was modified using catechol and polyamine. A poly(catechol-polyamine) (PCPA) layer was formed and deposited on the surface of silica microspheres as an adherent coating. Silver-coated silica microspheres were produced through the functionalization of the PCPA surface and electroless silver plating. The microspheres are then thermally treated to increase their electrical conductivity. The resulting SiO_2_/PCPA/Ag microspheres were incorporated into silicone rubber to create highly conductive rubber. The chemical composition, crystal structure, morphology, and electrical resistivity of the prepared samples were analyzed.

## 2. Materials and Methods

### 2.1. Materials

Silica microspheres with a imply particle measurement of 30 μm had been provided by means of Qinhuang Glass Microsphere Ltd. (Qinhuangdao, China). The silica microspheres have been ultrasonically cleaned in ethanol and deionized water for 30 min and dried in vacuum at 60 °C before use. Catechol, diethylenetriamine (DETA), triethylenetetramine (TETA), and tetraethylenepentamine (TEPA) were purchased from Tianjin Fuchen Chemical Reagents Factory (Tianjin, China). Tris(hydroxymethyl)-aminomethane (Tris), polyvinyl pyrrolidone (PVP), Silver nitrate, glucose, ammonia, ethanol, and hydrochloric acid had been acquired from Beijing Chemical Plant (Beijing, China). Methyl-vinyl silicone rubber (MVQ) was furnished by means of Zhonghao Chenguang Research Institute of Chemical Industry (Beijing, China). All commercially accessible reagents and solvents have been used as acquired and barring similarly purification.

### 2.2. Preparation of PCPA Modified Silica Microspheres

The 4 g of silica microspheres were immersed in Tris-HCl buffer solution (pH = 8.5, 10 mM) containing catechol and polyamine (10 mM) with the molar ratio of 3:1 under stirring for 24 h. Then, the PCPA coated silica were taken out, rinsed with deionized water thoroughly, and dried at 60 °C in vacuum. The PCPA coated silica microspheres were denoted as SiO_2_/PCPA.

### 2.3. Electroless Plating of Silver on the SiO_2_/PCPA Surface

A silver plating solution was prepared by gradually adding ammonia dropwise to a 100 mL solution of silver nitrate (10 g/L) until the solution became transparent. Subsequently, 0.25 wt% PVP was introduced to the silver-plating solution and stirred magnetically for 5 min. PVP acted as a dispersant and stabilizing agent. The 4 g SiO_2_/PCPA was immersed in the solution and stirred magnetically for 25 min. Following this, a 100 mL glucose (20 g/L) solution was added as the reducing agent. The resulting mixture was stirred for 60 min at room temperature before being filtered to separate the sample. The sample was thoroughly rinsed with deionized water and dried at 60 °C under vacuum for 6 h. The obtained sample was denoted as SiO_2_/PCPA/Ag. Finally, the SiO_2_/PCPA/Ag microspheres were subjected to heat treatment in a nitrogen-protected Muffle furnace at 400 °C for 30 min.

### 2.4. Fabrication of SiO_2_/PCPA/Ag Filled Silicone Rubber

The SiO_2_/PCPA/Ag microspheres were then blended with MVQ and vulcanizing agent 2,5-Dimethyl-2,5-di(tert-butylperoxy) hexane on a 6-inch two-roll mill. The rubber composites were preformed into sheet materials and underwent an initial stage of vulcanization using a vulcanizing press at a temperature of 170 °C and pressure of 10 MPa for a duration of t_90_ times. Then, the rubber composites underwent a 2nd stage of vulcanization in an oven at 200 °C for 2 h. The vulcanized rubber composites were laid apart for 8 h before property characterization. The obtained samples are denoted as SiO_2_/PCPA/Ag/MVQ.

### 2.5. Characterization

XPS was used to analyze the chemical composition of the sample surface using an XPS machine (ESCALAB 250, Thermo Electron Corporation, Waltham, MA, USA) with an Al Kα X-ray source at 150 W. Powdered samples were pressed into tablets and mounted on sample studs with adhesive tape. Binding energies were referenced to the C1s hydrocarbon peak at 284.6 eV to account for surface charging effects. Surface morphology was analyzed using a scanning electron microscope (SEM, S-4800, Hitachi, Tokyo, Japan), with samples mounted on pattern studs using adhesive tape. Platinum was sputtered onto the samples before measurement, and the SEM was operated at 20 kV. The samples’ crystalline shape was analyzed using Powder X-ray diffraction (XRD, D/Max2500VB2+/PC, Rigaku, Tokyo, Japan) with Cu Kα radiation, and the diffraction patterns have been recorded in reflection mode with the 2θ vary of 5−90°. The electrical resistivity of the samples was measured using a powder resistivity tester (FT-301 Ningbo ROOKO instrument Co., Ltd., Ningbo, China) through the four-terminal method. The SiO_2_/PCPA/Ag was pressed into a 1 mm sheet at 10 MPa pressure. The electromagnetic interference (EMI) shielding effectiveness of the rubber composites was determined using scattering parameters (S-parameters, specifically S_11_, S_21_, S_12_, and S_22_). S-parameters were measured by a vector network analyzer (N5244A, Agilent, Santa Clara, CA, USA). The S parameters were tested using waveguide method in the range of 5.4~8.2 GHz (C-band), 8.2~12.4 GHz (X-band), and 12.4~18.0 GHz (Ku-band). Regarding the size of the samples, they were cut into shapes of 15.75 mm × 34.70 mm, 22.90 mm × 10.20 mm, and 15.90 mm × 8.03 mm and measured 2 mm in thickness. The experimental setup included the connection of test cables, adapters, and appropriate waveguide test fixtures. Calibration was executed with the 31101 calibration kit. Following calibration, the test sample was positioned within the fixture, and its thickness was recorded. Measurements were conducted using rectangular waveguides operating in the TE10 mode of wave propagation.

## 3. Results and Discussion

### 3.1. PCPA Coordination on the Surface of SiO_2_ Microspheres

**Scheme 1:**
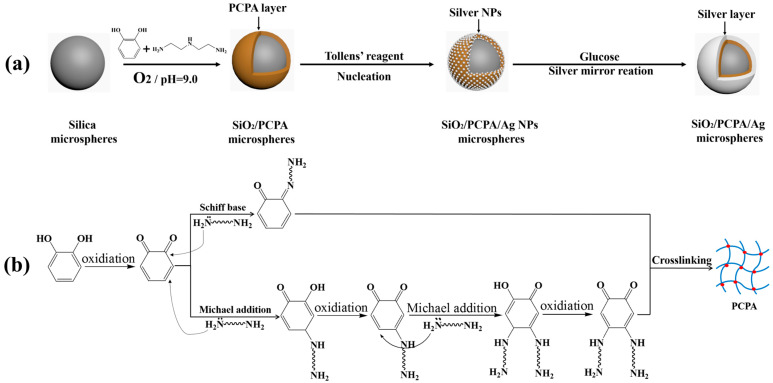
(**a**) Illustration of procedure for preparing silver-coated silica microspheres and (**b**) the possible reaction mechanism of catechol and polyamine.

The methodology for fabricating SiO_2_/Ag core–shell structured microspheres through PCPA-assisted electroless silver plating is illustrated in Scheme 1a. Initially, the silica microspheres were dispersed in an alkaline aqueous solution containing catechol and polyamine and stirred for 4 h to produce SiO_2_/PCPA microspheres. Subsequently, the SiO_2_/PCPA microspheres were submerged in a silver plating bath. The metallic binding potential of catechol group and the reducing capability of the amino group in PCPA facilitated the reduction of [Ag(NH_3_)^2^]^+^ ions to silver nanoparticles, which were then deposited on the surface of silica. The silver particles serve as nucleation sites during the electroless plating process, leading to the growth of a uniform and compact silver layer upon the addition of glucose. The mechanism of oxidation polymerization involving catechol and polyamine is illustrated in Scheme 1b. In an alkaline buffer solution, catechol is oxidized to form 1,2-benzoquinone, which then readily reacts with polyamine through two pathways: Schiff base reaction and Michael addition. The 1,2-benzoquinone undergoes Schiff base reaction with polyamine to yield 2-N-substituted-1,2-benzoquinone. Additionally, 1,2-benzoquinone reacts with polyamine via Michael addition to produce 4-N-substituted-1,2-benzoquinone, which further reacts with polyamine through Michael addition to yield 4,5-N, N-disubstituted-1,2-benzoquinone [29]. The polymerization of these resultant small molecular compounds leads to the formation of cross-linked polymer PCPA. The primary amine in polyamine exhibits greater reactivity compared to secondary amine, leading to preferential reaction of the terminal amine with catechol, while ethylene imine serves as a spacer group. Consequently, the cross-linking density of the polymer network can be modulated by adjusting the chain length of the ethylene imines [29].

### 3.2. Electroless Plating of Silver on the Surface of SiO_2_/PCPA

**Figure 1:**
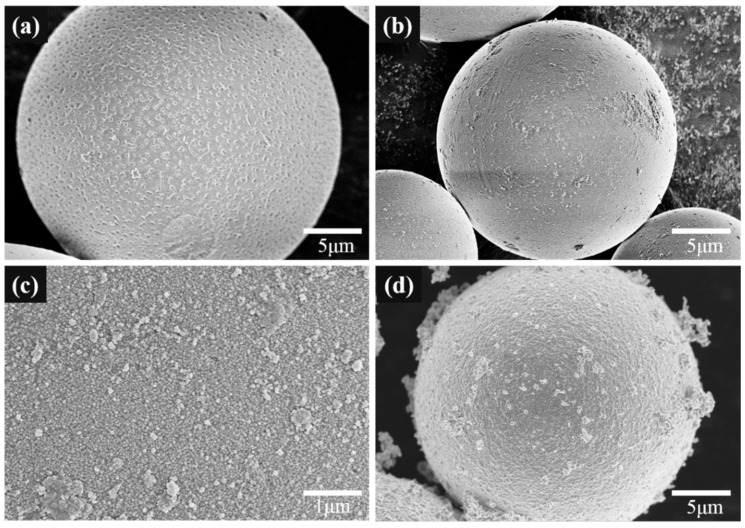
SEM images of (**a**) the pristine silica microspheres, (**b**) SiO_2_/PCPA microspheres, silver-coated SiO_2_/PCPA microspheres (**c**) without glucose and (**d**) with glucose.

Figure 1 displays scanning electron microscopy (SEM) images depicting the pristine silica surface in various conditions. We can observe a rough surface with numerous pores on the pristine silica microspheres (Figure 1a). Following modification the of PCPA, the pores on the pristine silica surface are eliminated, resulting in a homogeneous polymer coating (see Figure 1b). The surface of SiO_2_/PCPA microspheres features a significant presence of ortho-phenolic hydroxyl groups and amino groups. Notably, the ortho-phenolic hydroxyl group exhibits chelating properties towards silver ions, while the amino group displays mild reducibility, facilitating the reduction of silver ions to silver nanoparticles for anchoring onto the SiO_2_/PCPA surface. The PCPA layer serves a dual role in facilitating the reduction of silver ions and enhancing the adhesion of silver particles to the substrate. In the absence of glucose, the larger spacing between silver particles hinders the formation of a conductive path (Figure 1c), resulting in poor conductivity of 4 S/cm. However, the addition of glucose promotes the nucleation and growth of silver nanoparticles, leading to a more uniform and dense silver layer (Figure 1d) with improved conductivity of 1000 S/cm.

**Figure 2:**
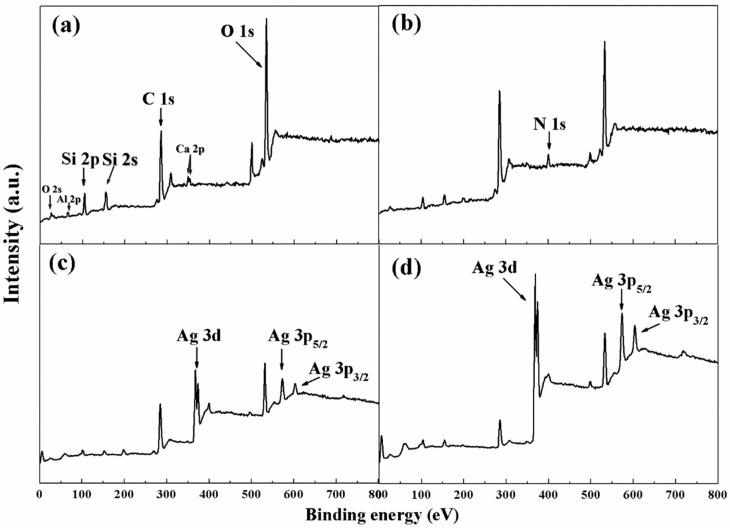
XPS wide scan spectra of (**a**) pristine silica, (**b**) SiO_2_/PCPA, silver-coated SiO_2_/PCPA microspheres (**c**) without glucose and (**d**) with glucose.

X-ray photoelectron spectroscopy (XPS) was utilized to analyze the surface chemical compositions of the samples. The wide-scan spectrum of the pristine silica microspheres is presented in Figure 2a. The C 1s core-level peak, observed at approximately 284.8 eV, is routinely employed for binding energy calibration. The peaks at 104 eV, 154.8 eV, and 532.9 eV correspond to the Si 2p, Si 2s, and O 1s photoelectron peaks of the silica microspheres, respectively. Additionally, the spectrum reveals other photoelectron and Auger peaks associated with the glass microspheres, including O 2s (26 eV), Al 2p (65 eV), Ca 2p (348.6 eV and 353.7 eV), Ca LMM Auger (307.6 eV), and O KLL Auger (499.5 eV). A new peak, N 1s, is evident in the wide-scan spectrum of SiO_2_/PCPA microspheres. The presence of the N element in polyamine is responsible for the ethylene imine. The observed Ag 3d peak at a binding energy of approximately 370 eV (Figure 2c) suggests the deposition of silver onto the SiO_2_/PCPA microspheres. Upon addition of glucose, a more pronounced Ag 3d peak is observed (Figure 2d), indicating a higher concentration of silver particles reduced on the surface of the SiO_2_/PCPA, consistent with the findings from SEM.

**Figure 3:**
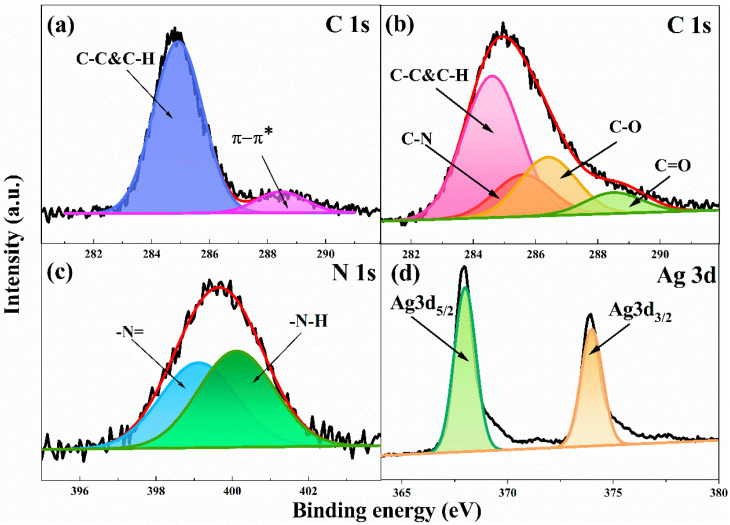
C 1s spectra of (**a**) pristine silica and (**b**) SiO_2_/PCPA, (**c**) N 1s spectra of SiO_2_/PCPA, (**d**) Ag 3d core-level spectra of SiO_2_/PCPA/Ag microspheres.

The XPS spectra were analyzed using Shirley background subtraction and fitted with Voigt profiles, which are a mixture of 70–80% Gaussian and 20–30% Lorentzian functions, optimized through nonlinear least-squares fitting. The Avantage v5.992 software was used with a Levenberg–Marquardt algorithm to minimize Δχ^2^ until it was less than 1 × 10^−4^. Specific constraints included a fixed C 1s peak at 284.8 eV with allowable shifts of ±0.2 eV for C-O/C-N and C=O peaks, and a FWHM less than 1.6 eV. For the N 1s peak, the -NH- main peak was fixed at 399.6 eV ± 0.2 eV, and for Ag 3d, the area ratio 3d_5_/_2_:3d_3_/_2_ was set at 3:2 with a spin–orbit splitting of 6.0 eV. The peak tails at ~372 eV and ~377 eV in Figure 3d arise from Ag MNN Auger transitions (M_4_N_45_N_45_ and M_5_N_45_N_45_). They are not photoemission peaks and were therefore excluded from the Ag 3d chemical-state fit. The C 1s core-level spectrum of pristine silica microspheres (Figure 3a) exhibits a single peak corresponding to C-C/C-H species at a binding energy (BE) of 284.8 eV, likely resulting from environmental contamination during sample preparation. In contrast, the C 1s core-level spectrum of SiO_2_/PCPA microspheres (Figure 3b) be curve-fitted five distinct peak components: C-H species at 284.8 eV, C-N species at 285.6 eV, C-O species at 286.6 eV, C=O species at 288.5 eV, and C=N species at 288.9 eV [30,31,32]. The presence of C=O species is indicative of catechol oxidation to quinone, while the C=N species is associated with the Schiff base. Following the modification of poly(catechol-polyamine) (PCPA), the characteristic peaks of N 1s become evident, with partial peak fitting revealing peaks at 399.5 eV (-N-) and 398.5 eV (-H-N-) resulting from the =N-group in the Schiff base reaction (Figure 3c). These findings confirm the polymerization mechanism of catechol and polyamine as outlined in Scheme 1, as well as the successful deposition of PCPA onto silica microspheres. Subsequent to the deposition of silver onto SiO_2_/PCPA, the Ag 3d core level exhibits two peaks at 368.0 eV and 374.0 eV for Ag 3d_5/2_ and Ag 3d_3/2_ binding energies, respectively (Figure 3d). It should be noted that these two peaks correspond to Ag_0_ species, which further confirms the existence of metallic Ag in the sample.

**Figure 4:**
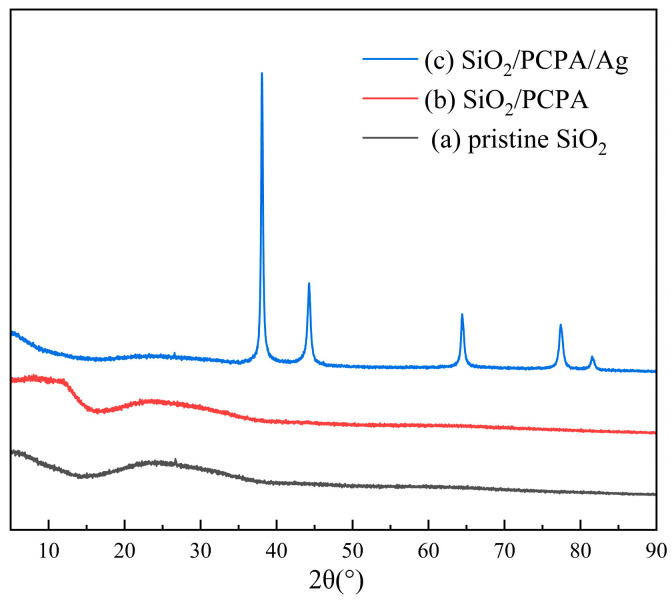
XRD patterns of (**a**) pristine silica microspheres, (**b**) SiO_2_/PCPA, and (**c**) SiO_2_/PCPA/Ag.

The determination of the crystalline shape of samples was conducted using XRD analysis. The significant diffuse peak observed at a 2θ angle of 24° in Figure 4a is attributed to the presence of amorphous pristine silica microspheres. Comparison between Figure 4a and Figure 4b reveals minimal differences, suggesting that the PCPA layers do not impact the crystalline structure of the silica microspheres. In Figure 4c, five distinct characteristic peaks are observed at 2θ values of 38.2°, 44.4°, 64.6°, 77.4°, and 81.6°, corresponding to the (1 1 1), (2 0 0), (2 2 0), (3 1 1), and (2 2 2) planes of FCC segment silver, respectively (JCPDS Card No. 4-783). The amorphous diffraction peak of SiO_2_ at 24° in the patterns becomes smoother. No diffraction peaks corresponding to silver halide or silver oxide were detected, suggesting that the silver deposited on the SiO_2_/PCPA substrate is in its elemental form. This finding is consistent with the XPS analysis.

**Figure 5:**
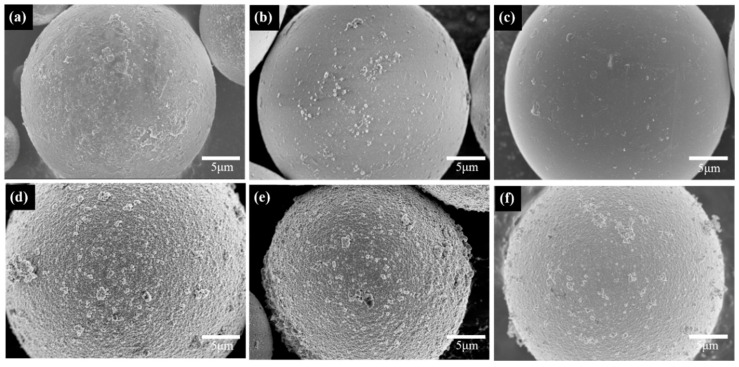
SEM images of the silica microspheres modified by catechol and different polyamines—(**a**) DETA, (**b**) TETA, (**c**) TEPA—and the silver-coated silica microspheres modified by catechol and different polyamines—(**d**) DETA, (**e**)TETA, and (**f**) TEPA.

The surface morphologies of the samples were examined using SEM. Figure 5 displays the SEM images of the SiO_2_/PCPA microspheres prepared with different polyamines: DETA, TETA, and TEPA. Following the modification of PCPA, the surface irregularities on the silica particles were eliminated. Instead of the presence of pores, the surface of silica is characterized by the presence of numerous nanoparticles when utilizing DETA (Figure 5a), whereas a thin film is observed on the surface of silica when employing TETA and TEPA (Figure 5b,c). The morphology of PCPA is impacted by the length of the polyamine, specifically the quantity of ethylene imine. The cross-linking density of the polymer matrix can be modified by varying the ethylene imine content, resulting in a high distribution density of benzene in PCPA when utilizing DETA with a shorter chain length. This high distribution density may lead to the stacking and aggregation of oligomers, ultimately leading to the formation of nanoparticles. The utilization of TEPA with a long chain resulted in a decrease in cross-linking density, leading to the formation of a thin film on the surface of silica microspheres. In comparison to DETA and TETA, the SiO_2_/PCPA/Ag utilizing TEPA (Figure 5f) exhibited a denser and more compact silver layer, attributed to the rougher surface facilitating the interlocking of the silica substrate with the silver nanoparticles during the electroless plating process. Considering the electrical resistivity of SiO_2_/PCPA/Ag, TEPA was selected for further experimentation.

**Figure 6:**
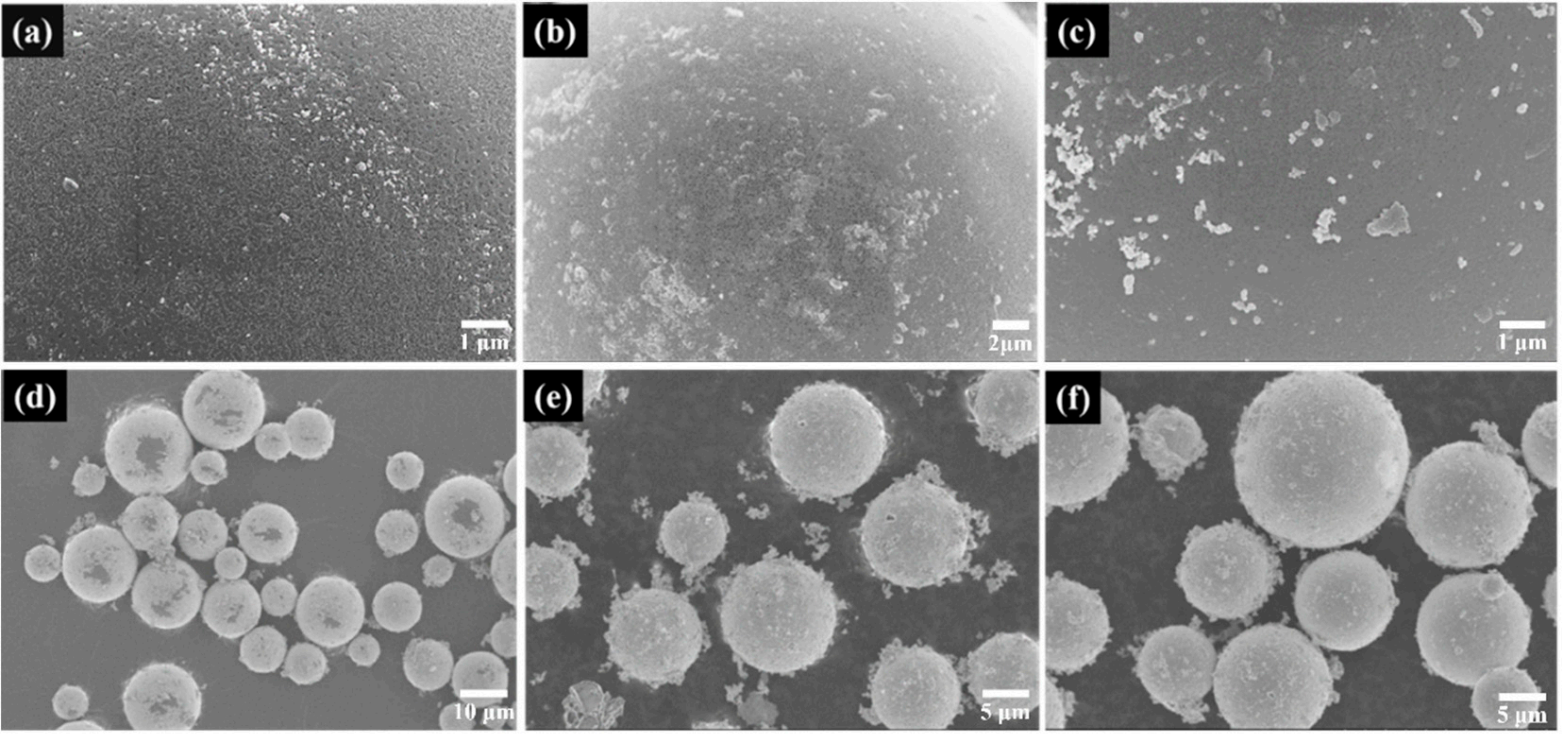
SEM images of SiO_2_ and SiO_2_/Ag microspheres modified by catechol/TEPA with different modification times: (**a**,**d**) 2 h, (**b**,**e**) 4 h, and (**c**,**f**) 6 h.

Figure 6a demonstrates that a catechol/TEPA modification time of 2 h results in decreased PCPA deposition on silica microspheres, which consequently leads to increased silver loss from the surface of the silica microspheres (Figure 6d). When the catechol/TEPA modification time is extended to 4 h, a uniform and complete silver layer is observed on the surface, suggesting that the modification process can achieve uniform and complete PCPA deposition on the silica microspheres after this duration (Figure 6b,e). The conductivity of the SiO_2_/PCPA/Ag microspheres has been measured at 1005 S/cm, meeting the required specifications and representing an 83% reduction in modification time compared to the dopamine modification method, which requires 24 h. Further analysis indicates that extending the catechol/TEPA modification time beyond 4 h does not significantly alter the surface of SiO_2_/PCPA/Ag (Figure 6c,f), with the optimal catechol/TEPA modification time identified as 4 h.

**Figure 7:**
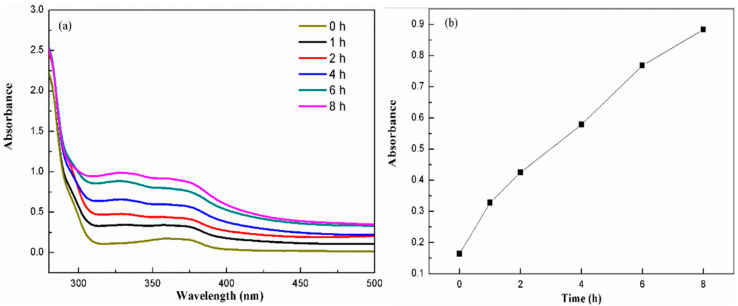
(**a**) UV-visible absorption spectra of catechol/TEPA solutions with different reaction times. (**b**) Effect of absorbance of catechol/TEPA solutions at 370 nm on different reaction times.

To evaluate the progression of the reaction between catechol and TEPA over time, an aqueous solution was prepared with catechol and TEPA (10 mM) with a molar ratio of 3:1. The pH was adjusted to 8.5 utilizing a Tris-HCl buffer, and the solution was subjected to magnetic stirring. Aliquots were extracted at intervals of 0, 1, 2, 4, 6, and 8 h, subsequently diluted, and analyzed for absorbance variations using a UV-Vis spectrophotometer. Figure 7 illustrates the UV-visible absorption spectra of the catechol/TEPA solution at various reaction intervals. The absorption peak observed at 370 nm serves as an indicator of the oxidation of catechol and o-benzoquinone during its interaction with a phenolic amine. As the reaction advances, the absorption intensity at 370 nm in the UV-visible spectrum progressively increases, signifying an enhancement in the reaction extent between catechol and TEPA (Figure 7a). The absorption intensity of the catechol/TEPA system at 370 nm exhibits an approximately linear increase over time, with a diminishing growth rate, suggesting that the reaction is approaching completion (Figure 7b).

**Figure 8:**
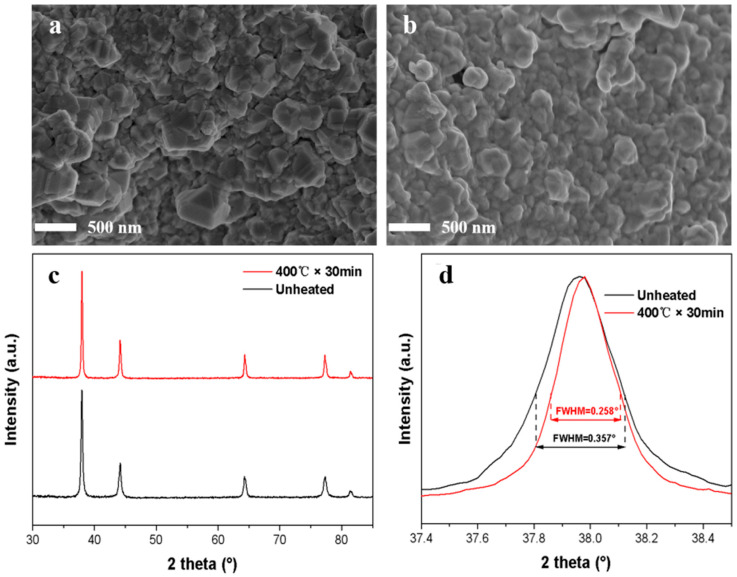
SEM images of SiO_2_/PCPA/Ag with (**a**) pre-heat treatment and (**b**) after heat treatment. XRD spectra of SiO_2_/PCPA/Ag with (**c**,**d**).

To enhance the electrical conductivity of SiO_2_/PCPA/Ag, a heat treatment was performed in a muffle furnace at 400 °C for 30 min. The results of this process are presented in Figure 8 and Table 1. The data indicate that the crystalline structure of the silver layer remains unchanged before and after the heat treatment (Figure 8c). However, there is a significant improvement in electrical conductivity, which increased from 1005 S/cm to 1612 S/cm, representing a 60.4% increase (Table 1). The optimal heat treatment condition for these microspheres is identified as 30 min at 400 °C. At a lower temperature of 200 °C, a prolonged duration of 2 h is required to achieve comparable electrical conductivity. Conversely, increasing the temperature to 500 °C results in the oxidation of elemental silver, leading to a significant reduction in electrical conductivity. Prior to heat treatment, the interstitial spaces between silver grains on the surface of silver-coated silica microspheres are relatively large (Figure 8a), impeding the movement of free electrons and consequently resulting in reduced electrical conductivity. Upon subjecting the SiO_2_/PCPA/Ag composite to heat treatment, a marked enhancement in the smoothness of the silver layer surface is evident (Figure 8b). During this process, a portion of the silver melts, and upon cooling, the molten silver acts as a binding agent, effectively filling the gaps between silver crystals. Analysis of the changes in the diffraction peak of the crystal indicates a reduction in the half peak width of the silver layer crystal’s diffraction peak from 0.357° to 0.258° post-heat treatment (Figure 8d). The heat treatment results in an increase in Ag grain size, as demonstrated by the full width at half maximum (FWHM) of the peak, which is related to particle size through the Scherrer equation. As the particle size increases, the number and size of intergrain interfaces decrease. Consequently, free charges experience less interface scattering, leading to an observed increase in conductivity.

### 3.3. Fabrication of Silicone Rubber Composites Filled with SiO_2_/PCPA/Ag Microspheres

**Figure 9:**
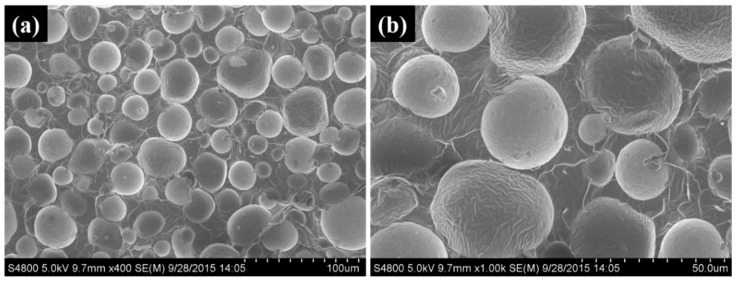
(**a**,**b**) Cross-sectional SEM images of SiO_2_/PCPA/Ag/MVQ.

The dispersion of SiO_2_/PCPA/Ag within silicone rubber was examined using SEM. The analysis revealed that the SiO_2_/PCPA/Ag microspheres are uniformly distributed throughout the silicone rubber matrix, exhibiting no visible aggregation. Furthermore, the microspheres are well-integrated with the silicone rubber filled with 250 phr SiO_2_/PCPA/Ag microspheres, with no observable gaps or voids (refer to Figure 9a). Despite undergoing a rigorous shearing process during rubber processing and high-temperature vulcanization, the silver layer on the surface of the SiO_2_/PCPA/Ag microspheres remains intact, suggesting a strong adhesion between the silver layer and the microspheres. Subsequent evaluations of the composite material’s physical properties demonstrated a tensile strength of 2.1 MPa and an elongation at break of 115%. Additionally, the volume resistivity of the composite material was measured at 5.4 × 10^−3^ Ω·cm, which increased to 7.1 × 10^−3^ Ω·cm after aging at 200 °C for 48 h. These findings indicate that the SiO_2_/PCPA/Ag/MVQ composite material synthesized in this study possesses significant conductivity and mechanical properties, making it suitable for applications in electromagnetic shielding.

When an electromagnetic wave interacts with an electromagnetic-shielding material, it typically undergoes three conversion processes: reflection, absorption, and transmission [33]_._ Reflection involves the propagation of the electromagnetic wave in media with different impedances, leading to a gradual weakening or complete dissipation of the wave, which results in reflection loss. Absorption is influenced by dielectric loss, magnetic loss, and conduction loss. These processes are categorized into three types based on their loss mechanisms: (1) reflection shielding (SE_R_), (2) absorption shielding (SE_A_), and (3) multiple reflection loss (SE_M_). According to Schelkunoff’s theory [34], the total electromagnetic shielding efficiency (SE_T_) is the cumulative effect of reflection losses, absorption losses, and multiple reflection losses. Multiple reflection effect can be safely neglected (SE_M_ ≈ 0) when material thickness is bigger than skin depth or absorption loss (SE_A_) is higher than 10 dB since the amplitude of the EM wave firstly reaching the second interface is negligible [35]. From the perspective of electromagnetic energy, when the EM wave is incident on a material, the incident power is divided into the reflected power, absorbed power and transmitted power. The corresponding power coefficients of reflectivity(R), absorptivity (A) and transmissivity(T) follow the law of power balance (R +A + T = 1) [36,37,38]. Scattering parameters, such as S_11_, S_12_, S_21_, and S_22_, are measured using a vector network analyzer (VNA).

T = |S_12_|^2^ = |S_21_|^2^ (1)

R = |S_11_|^2^ = |S_22_|^2^ (2)

A = 1 − R − T(3)

SE_R_ = −10log(1 − R) (4)

SE_A_ = −10log(T/1 − R) (5)

SE_T_ = SE_R_ + SE_A_ + SE_M_ (6)

**Figure 10:**
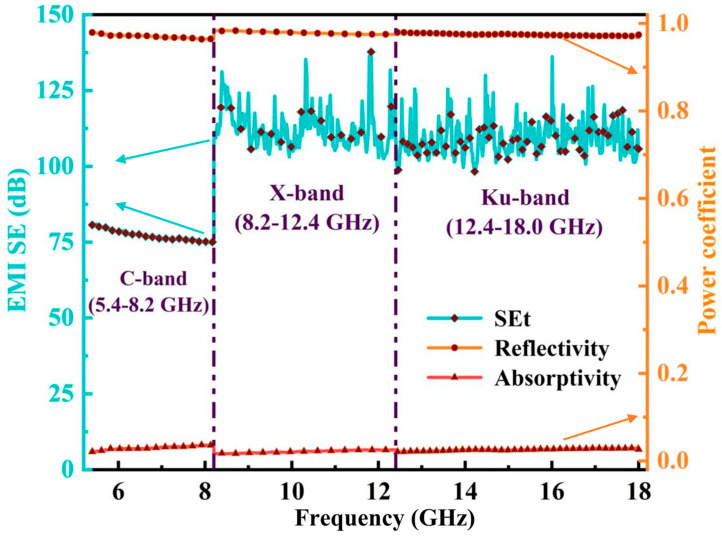
EMI shielding effectiveness and power coefficient of SiO_2_/PCPA/Ag/MVQ composite in C, X and Ku-band.

The SiO_2_/PCPA/Ag/MVQ composite material, with a thickness of 2 mm, exhibits a total electromagnetic shielding effectiveness (SE_T_) exceeding 75 dB in the C-band and surpassing 100 dB in the X-band and Ku-band (Figure 10). This level of performance corresponds to an attenuation of more than 99.99999999% of electromagnetic waves, significantly surpassing the military standard for electromagnetic shielding rubber, which is set at a minimum of 60 dB. When electromagnetic waves impinge upon the surface of a highly conductive rubber, the material’s high density of free electrons induces a surface current, which in turn generates an induced magnetic field opposing the external electromagnetic waves. This process results in the reflection of a significant portion of the electromagnetic waves. The waves that are not reflected penetrate the material’s interior, where their energy is absorbed by the free electrons within the conductive filler. This energy absorption converts the electromagnetic energy into electronic kinetic energy and thermal energy, thereby diminishing the intensity of the electromagnetic waves. Within the three-dimensional network formed by the conductive filler, the electromagnetic waves undergo multiple reflections and scattering at the particle interfaces, with each reflection accompanied by energy loss, thereby enhancing the material’s shielding effectiveness. Although reflection loss and absorption loss describe reflection and absorption, respectively, in both Schelkunoff’s theory and calculation theory, they do not accurately represent the actual levels of reflected and absorbed power [39]. It is reasonable and intuitive to adopt power coefficients of R and A to determine the type of shielding materials and shielding mechanisms [40,41]. If R is higher than A, reflection is the dominant shielding mechanism. The reflectivity (R, orange line) of the composite material reaches 0.96 within the frequency range of 5.4–18.0 GHz, suggesting that reflection loss is the primary mechanism of electromagnetic shielding. The substantial impedance mismatch encountered as electromagnetic waves transition from air to the conductive rubber surface results in the reflection of the majority of these waves. Further, Table 2 presents the electromagnetic shielding effectiveness of silver-coated microspheres/polymer composites reported in recent years. Notably, the silver-coated glass microspheres/MVQ composite fabricated in this study exhibited the highest shielding effectiveness across a broader frequency range. The outstanding electromagnetic shielding performance across such a broad frequency spectrum renders this composite material highly suitable for applications in electromagnetic shielding, particularly in domains such as meteorological radar, fire control radar, air defense radar, satellite communication, and remote sensing and mapping.

## 4. Conclusions

In conclusion, we utilized a biomimetic modification approach involving the co-deposition of catechol and polyamine to alter the properties of silica microspheres. The optimal modification was achieved with a molar ratio of tetraethylenepentamine to catechol of 1:3 and a reaction duration of 4 h, rendering the process both cost-effective and efficient. Following this, silver-coated silica microspheres (SiO_2_/PCPA/Ag) were synthesized via electroless silver plating. Post heat treatment at 400 °C for 30 min, the electrical conductivity of these silver-plated microspheres reached up to 1612 S/cm. When incorporated into silica rubber with 250 phr SiO_2_/PCPA/Ag, the resulting composite exhibited a low resistivity of 5.4 × 10^−3^ Ω·cm and an electromagnetic shielding effectiveness exceeding 100 dB across both the X-band and Ku-band, indicating superior electromagnetic shielding capabilities. The primary mechanism underlying the electromagnetic shielding of this composite material is reflection, making it highly suitable for applications in satellite communication, radar, remote sensing, and mapping, among other fields.

## Figures and Tables

**Table 1:** Electrical conductivity of SiO_2_/PCPA/Ag before and after heat treatment.

Sample	Electrical Conductivity (S/cm)	Electrical Resistivity (Ω·cm)
Pre-heat treatment	1005	0.995 × 10^−3^
200 °C × 30 min	1029	0.972 × 10^−3^
200 °C × 2 h	1466	0.682 × 10^−3^
300 °C × 30 min	1044	0.958 × 10^−3^
300 °C × 1.5 h	1513	0.661 × 10^−3^
400 °C × 30 min	1612	0.620 × 10^−3^
500 °C × 30 min	524	1.908 × 10^−3^

**Table 2:** Comparison of electromagnetic SE of silver-coated microspheres/polymers.

Sample	Substrate	Filler Content	Thickness (mm)	Frequency (GHz)	SE (dB)	Ref.
Ag/GM	PDMS	77.7 wt%	2	8.2–12.4	100	[42]
Ag/GM	epoxy		-	8.2–12.4	50	[43]
Carbon fiber + Ag@HGMs	epoxy	15 wt% + 50 wt%	1	8.2–12.4	88.1	[44]
Ag@HGM + short carbon fiber	epoxy	1 wt% + 5 wt%	2	8.2–12.4	34.6	[45]
Ag/HGM	epoxy resin adhesive	50 vol%	-	2.0–12.0	75	[24]
Al/Ag	MVQ	71.4 wt%	2	8.2–12.4	70	[29]
Ag@HGM + Fe_3_O_4_@CNT	silicone rubber	0.51 vol% + 1.6 vol%	2	8.2–12.4	59.4	[46]
Ag/hollow carbon microspheres	epoxy resin	30.5 wt%	1.5	8.2–12.4	60.2	[47]
Ag/GM	MVQ	71.4 wt%	2	5.4–8.2	75	This work
Ag/GM	MVQ	71.4 wt%	2	8.2–18.0	100	

## References

[1] Wang Y.Q., Zhao W., Tan L.L., Li Y.R., Qin L., Li S.D. (2023). Review of polymer-based composites for electromagnetic shielding application. Molecules.

[2] Chung D.D.L. (2020). Materials for electromagnetic interference shielding. Mater. Chem. Phys..

[3] Yu L.J., Yang Q.X., Liao J.L., Zhu Y.F., Li X., Yang W.T., Fu Y.Q. (2018). A novel 3D silver nanowires@polypyrrole sponge loaded with water giving excellent microwave absorption properties. Chem. Eng. J..

[4] Cheng Y., Zhu W.D., Lu X.F., Wang C. (2022). One-dimensional metallic, magnetic, and dielectric nanomaterials-based composites for electromagnetic wave interference shielding. Nano Res..

[5] Daoush W.M., El-Tantawy A., Morsi K., El-Nikhaily A.E. (2024). Novel cubic boron nitride-reinforced Cu/Ni alloy elemental metal matrix composites for electromagnetic radiation shielding applications. J. Mater. Eng. Perform..

[6] Huang D.Y., Wu M., Kuga S., Huang Y. (2022). Graphite nanosheet-based carbon foams for electromagnetic interference shielding. ACS Appl. Nano Mater..

[7] Kumar R., Sahoo S., Joanni E., Singh R.K., Tan W.K., Kar K.K., Matsuda A. (2021). Recent progress on carbon-based composite materials for microwave electromagnetic interference shielding. Carbon.

[8] Ma M., Tao W.T., Liao X.J., Chen S., Shi Y.Q., He H.W., Wang X. (2023). Cellulose nanofiber/MXene/FeCo composites with gradient structure for highly absorbed electromagnetic interference shielding. Chem. Eng. J..

[9] Pan Y.F., Dai M.Y., Guo Q., Yin D.W., Hu S.Q., Hu N.G., Zheng X., Huang J.T. (2023). Construction of sandwich-structured Cu-Ni wood-based composites for electromagnetic interference shielding. Chem. Eng. J..

[10] Li H.Y., Ru X.H., Song Y., Wang H.P., Yang C.H., Zheng S.R., Gong L., Zhang X.G., Duan H.J., Liu Z.G. (2022). Flexible sandwich-structured silicone rubber/MXene/Fe_3_O_4_ composites for tunable electromagnetic interference shielding. Ind. Eng. Chem. Res..

[11] Du Y., Dai Z., Bu F., Li M., Li G., Liu L., Wang J., Xu C., Zhang J. (2023). Preparation and properties of flexible electromagnetic shielding composites. J. Mater. Sci. Mater. Electron..

[12] Wanasinghe D., Aslani F. (2019). A review on recent advancement of electromagnetic interference shielding novel metallic materials and processes. Compos. Part B Eng..

[13] Hicyilmaz A.S., Bedeloglu A.C. (2022). Electromagnetic interference shielding thermoplastic composites reinforced with carbon based hybrid materials: A review. Compos. Interfaces.

[14] Nan Z., Wei W., Lin Z.H., Chang J.J., Hao Y. (2023). Flexible nanocomposite conductors for electromagnetic interference shielding. Nano-Micro Lett..

[15] Wang Y., Huang J.T. (2017). Mechanical performance and electromagnetic shielding effectiveness of composites based on Ag-plating cellulose micro-nano fibers and epoxy. J. Polym. Eng..

[16] Cui K.X., Yan B., Xie Y.J., Qian H., Wang X.G., Huang Q.X., He Y.H., Jin S.M., Zeng H.B. (2018). Regenerable urchin-like Fe_3_O_4_@PDA-Ag hollow microspheres as catalyst and adsorbent for enhanced removal of organic dyes. J. Hazard. Mater..

[17] Liu S.Y., Liu L.H., Su G., Zhao L., Peng H.L., Xue J.R., Tang A.P. (2022). Enhanced adsorption performance, separation, and recyclability of magnetic core-shell Fe_3_O_4_@PGMA-g-TETA-CSSNa microspheres for heavy metal removal. React. Funct. Polym..

[18] Al-Bermany E., Mekhalif A.T., Banimuslem H.A., Abdali K., Sabri M.M. (2023). Effect of green synthesis bimetallic Ag@SiO_2_ core–shell nanoparticles on absorption behavior and electrical properties of PVA-PEO nanocomposites for optoelectronic applications. Silicon.

[19] Cai G., Ni H.S., Li X.Z., Wang Y.X., Zhao H.X. (2023). Eco-friendly fabrication of highly stable silica aerogel microspheres with core-shell structure. Polymers.

[20] Xu L.Q., Yap B.S.M., Wang R., Neoh K.G., Kang E.T., Fu G.D. (2014). Catecholamine-induced electroless metallization of silver on silica@polymer hybrid nanospheres and their catalytic applications. Ind. Eng. Chem. Res..

[21] Jundale R.B., Bari A.H., Kulkarni A.A. (2023). Insights into the synthesis and kinetics of silver-on-silica core-shell particles. Langmuir.

[22] Huang C.K., Chen C.Y., Han J.L., Chen C.C., Jiang M.D., Hsu J.S., Chan C.H., Hsieh K.H. (2010). Immobilization of silver nanoparticles on silica microspheres. J. Nanopart. Res..

[23] Ma Z.J., Jiang Y.W., Xiao H.S., Jiang B.F., Zhang H., Peng M.Y., Dong G.P., Yu X., Yang J. (2018). Sol-gel preparation of Ag-silica nanocomposite with high electrical conductivity. Appl. Surf. Sci..

[24] Huang Z., Chi B., Guan J.G., Liu Y.Q. (2014). Facile method to synthesize silver nanoparticles on the surface of hollow glass microspheres and their microwave shielding properties. RSC Adv..

[25] Whaley S.R., English D.S., Hu E.L., Barbara P.F., Belcher A.M. (2000). Selection of peptides with semiconductor binding specificity for directed nanocrystal assembly. Nature.

[26] Guo Z.D., Zhang D., Qiu H.L., Ju Y.M., Zhang T., Zhang L.J., Meng Y., Wei Y.J., Chen G. (2016). Improved electrochemical properties of tavorite LiFeSO_4_F by surface coating with hydrophilic poly-dopamine via a self-polymerization process. RSC Adv..

[27] Liu Y.L., Ai K.L., Lu L.H. (2014). Polydopamine and its derivative materials: Synthesis and promising applications in energy, environmental, and biomedical fields. Chem. Rev..

[28] Wang H., Wu J.J., Cai C., Guo J., Fan H.S., Zhu C.Z., Dong H.X., Zhao N., Xu J. (2014). Mussel inspired modification of polypropylene separators by catechol/polyamine for Li-ion batteries. ACS Appl. Mater. Interfaces.

[29] Hao M.Z., Li L., Shao X.M., Tian M., Zou H., Zhang L.Q., Wang W.C. (2022). Fabrication of highly conductive silver-coated aluminum microspheres based on poly(catechol/polyamine) surface modification. Polymers.

[30] Grey L.H., Nie H.Y., Biesinger M.C. (2024). Defining the nature of adventitious carbon and improving its merit as a charge correction reference for XPS. Appl. Surf. Sci..

[31] Feng X., Li R., Ma Y., Chen R., Shi N., Fan Q., Huang W. (2011). One-step electrochemical synthesis of graphene/polyaniline composite film and its applications. Adv. Funct. Mater..

[32] Taylor S., Hallböök F., Temperton R.H., Sun J., Rämisch L., Gericke S.M., Ehn A., Zetterberg J., Blomberg S. (2024). In situ ambient pressure photoelectron spectroscopy study of the plasma–surface interaction on metal foils. Langmuir.

[33] Wang M., Tang X.H., Cai J.H., Wu H., Shen J.B., Guo S.Y. (2021). Construction, mechanism and prospective of conductive polymer composites with multiple interfaces for electromagnetic interference shielding: A review. Carbon.

[34] Li J.W., Ding Y.Q., Yu N., Gao Q., Fan X., Wei X., Zhang G.C., Ma Z.L., He X.H. (2020). Lightweight and stiff carbon foams derived from rigid thermosetting polyimide foam with superior electromagnetic interference shielding performance. Carbon.

[35] Song W.L., Cao M.S., Lu M.M., Bi S., Wang C.Y., Liu J., Yuan J., Fan L.Z. (2014). Flexible graphene/polymer composite films in sandwich structures for effective electromagnetic interference shielding. Carbon.

[36] Gorakhpurwalla H.D., McGinty R.J., Watson C.A. (1975). Microwave dissipation loss in high-moisture grain. J. Agric. Eng. Res..

[37] Lin M.S., Chen C.H. (1993). Plane-wave shielding characteristics of anisotropic laminated composites. IEEE Trans. Electromagn. Compat..

[38] McDowell A.J., Hubing T.H. (2014). Analysis and comparison of plane wave shielding effectiveness decompositions. IEEE Trans. Electromagn. Compat..

[39] Song Q., Ye F., Yin X.W., Li W., Li H.J., Liu Y.S., Li K.Z., Xie K.Y., Li X.H., Fu Q.G. (2017). Carbon nanotube–multilayered graphene edge plane core–shell hybrid foams for ultrahigh-performance electromagnetic-interference shielding. Adv. Mater..

[40] Wan Y.J., Zhu P.L., Yu S.H., Sun R., Wong C.P., Liao W.H. (2018). Anticorrosive, ultralight, and flexible carbon-wrapped metallic nanowire hybrid sponges for highly efficient electromagnetic interference shielding. Small.

[41] Peng M., Qin F. (2021). Clarification of basic concepts for electromagnetic interference shielding effectiveness. J. Appl. Phys..

[42] Xu R., Sheng S., Hu C., Xie H., Huang B., Lou P., Fei H.F., Zhang Z. (2025). Lightweight flexible and efficient electromagnetic shielding composites based on silver-coated glass microspheres. ACS Omega.

[43] Wang Y., Ren K., Sun J., Li W., Zhao S., Chen Z., Guan J. (2017). Ultralow content silver densely-coated glass microsphere for high performance conducting polymer-matrix composites. Compos. Sci. Technol..

[44] Chen W., Qin Y., He X., Su Y., Wang J. (2021). Light-weight carbon fiber/silver-coated hollow glass spheres/epoxy composites as highly effective electromagnetic interference shielding material. J. Reinf. Plast. Comp..

[45] Thomas B., Parameswarreddy G., Sarathi R., Arunachalam K., Suematsue H., Verma R., Sharma A. (2024). Electromagnetic shielding effectiveness of silver-coated hollow glass microsphere-graded short carbon fiber epoxy composite. Polym. Compos..

[46] Yang J., Liao X., Wang G., Chen J., Guo F., Tang W., Wang W., Yan Z., Li G. (2020). Gradient structure design of lightweight and flexible silicone rubber nanocomposite foam for efficient electromagnetic interference shielding. Chem. Eng. J..

[47] Zhang L., Roy S., Chen Y., Chua E.K., See K.Y., Hu X., Liu M. (2014). Mussel-inspired polydopamine coated hollow carbon microspheres, a novel versatile filler for fabrication of high performance syntactic foams. ACS Appl. Mater. Interfaces.

